# Estimating Brazilian states’ demands for intensive care unit and clinical hospital beds during the COVID-19 pandemic: development of a predictive model

**DOI:** 10.1590/1516-3180.2020.0517.r1.0212020

**Published:** 2021-03-12

**Authors:** João Flávio de Freitas Almeida, Samuel Vieira Conceição, Luiz Ricardo Pinto, Cláudia Júlia Guimarães Horta, Virgínia Silva Magalhães, Francisco Carlos Cardoso de Campos

**Affiliations:** I PhD. Assistant Professor, Department of Industrial Engineering, Universidade Federal de Minas Gerais (UFMG), Belo Horizonte (MG), Brazil.; II PhD. Professor, Department of Industrial Engineering, Universidade Federal de Minas Gerais (UFMG), Belo Horizonte (MG), Brazil.; III PhD. Professor, Department of Industrial Engineering, Universidade Federal de Minas Gerais (UFMG), Belo Horizonte (MG), Brazil.; IV PhD. Science and Technology Researcher, Directorate of Public Policy, Fundação João Pinheiro (FJP), Belo Horizonte (MG), Brazil.; V MSc. Doctoral Student, Núcleo de Educação em Saúde Coletiva (NESCON), School of Medicine, Universidade Federal de Minas Gerais (UFMG), Belo Horizonte (MG), Brazil.; VI MSc. Public Health Physician, Núcleo de Educação em Saúde Coletiva (NESCON), School of Medicine, Universidade Federal de Minas Gerais (UFMG), Belo Horizonte (MG), Brazil.

**Keywords:** Public health administration, Bed occupancy, Coronavirus infections, Pandemics, Hospital bed capacity, Public health planning, Population dynamics, COVID-19 pandemic, COVID-19 virus disease, Compartmental model

## Abstract

**BACKGROUND:**

The fragility of healthcare systems worldwide had not been exposed by any pandemic until now. The lack of integrated methods for bed capacity planning compromises the effectiveness of public and private hospitals’ services.

**OBJECTIVES:**

To estimate the impact of the COVID-19 pandemic on the provision of intensive care unit and clinical beds for Brazilian states, using an integrated model.

**DESIGN AND SETTING:**

Experimental study applying healthcare informatics to data on COVID-19 cases from the official electronic platform of the Brazilian Ministry of Health.

**METHODS:**

A predictive model based on the historical records of Brazilian states was developed to estimate the need for hospital beds during the COVID-19 pandemic.

**RESULTS:**

The proposed model projected in advance that there was a lack of 22,771 hospital beds for Brazilian states, of which 38.95% were ICU beds, and 61.05% were clinical beds.

**CONCLUSIONS:**

The proposed approach provides valuable information to help hospital managers anticipate actions for improving healthcare system capacity.

## INTRODUCTION

The novel severe acute respiratory syndrome coronavirus 2 (SARS-CoV-2) disease^1^^–^^3^ was first reported in Wuhan, China, in December 2019. Since then, political leaders and healthcare managers have endeavored to estimate the demand for intensive care unit (ICU) and clinical beds to guard against the possibility of collapse of the healthcare system. Moreover, political leaders have adopted proactive non-pharmaceutical interventions (NPIs) to reduce fatalities arising from bed shortages. Although the quarantine and travel restrictions in Wuhan delayed progression of the epidemic on an international scale, thereby reducing case importations by nearly 80% until mid-February,^4^^–^^6^ the virus has since then spread rapidly both domestically and internationally.^5^^,^^7^^,^^8^ In Brazil, confirmed cases started to be seen in state capital municipalities and then moved towards non-metropolitan cities, and the numbers of cases continue to grow in every state.^9^^,^^10^

Brazil identified its first COVID-19 case on February 25, 2020, and its first death on March 17, 2020. A lack of consensus on public healthcare policy^11^^–^^12^ compromised use of integrated and coordinated NPIs to reduce COVID-19 mortality and healthcare demand. Moreover, the lack of integrated methods for bed capacity planning compromised the effectiveness of public and private hospitals’ services.

Mathematical models are used to evaluate the efficacy of specific interventions that were implemented in the past, in order to identify future strategies and effectively inform public health policy.^13^^,^^14^ The challenge in this work consists of forecasting the numbers of individuals requiring hospitalization and predicting the availability of hospital beds for such patients, taking into account the technical limitations of the data,^15^^–^^17^ infection rates and under-reporting of cases^18^ because of low numbers of tests. Therefore, we limited our analysis to the official data.^19^^,^^20^

Tertiary-care hospital beds are not equally available for all citizens. In Brazil, there are 2.4 beds/1,000 inhabitants and 34,318 ICU beds. 54% of the ICU beds are public, assigned to 78% of the population, while the remaining 22% of the population that has access to private care receive this medical care on 46% of the ICU beds.

In the following sections, we briefly describe the model and the estimates for the required numbers of ICU and clinical beds for each Brazilian state that would be needed to avoid healthcare system collapse.

## OBJECTIVES

The purpose of this paper was to present a combined approach to estimation of Brazilian states’ demands for ICU and clinical beds during a pandemic.

## METHOD

### ICU and clinical bed dynamics

Consider a compartmental model^21^^,^^22^ in which the population is divided into susceptible (S), exposed (E), infectious (I) and recovered (R) individuals (SEIR model). A rate of β of *S* individuals in contact with *I* (S-I contact) becomes E and progresses over the course of the incubation period at a rate σ, to state I. While a rate γ of I recovers from the disease, a rate μ_I_ of I evolves to death. A fraction ξ of R (recovered individuals) may become (or not, if ξ = 0) re-susceptible (S) and, therefore, a SEIRS model.

The effect of testing a segment of the population is modeled by introducing the rate of transmission for individuals with detected infection (β_D_), detected exposed state (D_E_) and detected infectious state (D_I_). Let ψ_E_ and ψ_I_ be the probabilities of positive tests for exposed and infected individuals, and Q, the rate of individuals with detected infection interacting with the population. Then D_E_ and D_I_ result from the rates θ_E_ψ_E_ and θ_I_ψ_I_ of testing exposed E and infected I individuals, respectively. The model describes the full spectrum of disease. Let N be the estimate of an affected population. Thus, N = S + E + I + D_E_ + D_I_ + R.

To represent the ICU and clinical bed dynamics, consider that a fraction of infected individuals is asymptomatic (I_A_). The rate α for symptomatic cases (I_S_ = I – I_A_) requiring hospitalization (H) is αI_S_. Let *T* be the number of planning days during the pandemic with *t* corresponding to each admission day at the hospital. For clinical bed dynamics, consider L_S_ and L_f_ as the average lengths of stay (LoS) of surviving patients and patients who died, respectively. For surviving patients in ICU beds, the average length of stay is L_b_ + L_d_ + L_a_, where L_b_ represents the surviving patients in clinical beds who are re-directed to ICU beds, L_d_ is the average LoS of surviving patients in ICU beds, and L_a_, the average LoS in clinical beds among surviving patients after being re-directed from ICU beds. For deceased victims of ICU bed dynamics, the average LoS is L_c_ + L_i_, representing the average periods for which a deceased patient will stay in clinical and ICU beds, respectively.

Given the average length of stay metrics for ICU and clinical dynamics, we calculate admission and leave days for each patient profile. For a surviving clinical patient, whose admission day is t, the expected day on which this patient leaves the clinical bed is T_o_ = t + L_S_ – 1. A clinical patient who progressed to death is expected to be removed from the hospital in T_f_ = t + L_f_ – 1. A surviving ICU patient is admitted in T_i_ = t + L_b_. In T_r_ = T_i_ + L_d_, the patient returns to the clinical bed, and in T_c_ = T_r_ + L_a_ – 1, the patient leaves the clinical bed. For a deceased ICU patient, the admission day is T_d_ = t + L_c_, and T_u_ = T_d_ + L_i_ – 1 is the expected day on which the patient is removed from the hospital.

Let H_t_ be the daily admission of patients to hospitals, and τ the fraction of hospitalized cases that require critical care in the ICU. Also, consider ζ and η to be the rates of critical patients who evolve to death in ICU beds and clinical beds, respectively.

**Figure 1:**
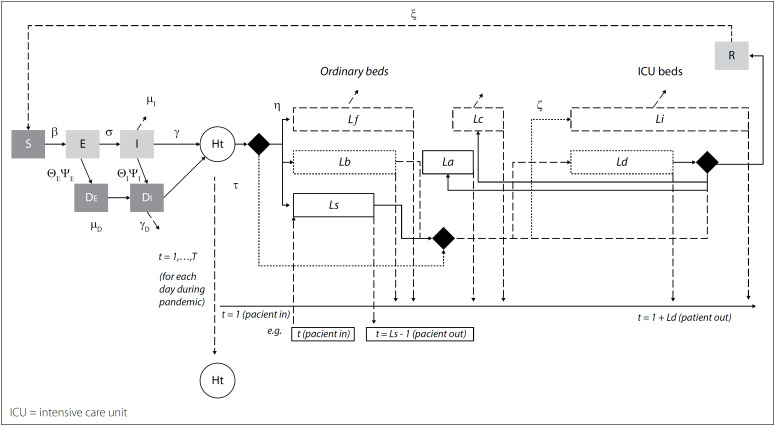
Model for susceptible (S), exposed (E), infectious (I), recovered (R) and re-susceptible (S) individuals (SEIRS) in Brazilian states: Pandemic model integrated with dynamic intensive care unit and clinical hospital beds.

The integrated model is presented in **Figure 1**. The model equations and data are available in a **Data Repository** that is available from https://github.com/joaoflavioufmg/webcovid19.

### Data sources and measurements

The model data included the social distancing index, which ranges from 30%, as observed at earlier times in the COVID-19 pandemic, to 100% in a possible lockdown situation.^23^ The latter would be expected to reduce the transmission rate of the model by 74%.^24^ Furthermore, the basic reproduction number, which is the average number of secondary cases generated per case, was set to 2.9 in the case of the Brazilian outbreak.^15^ After the first month, we set customized transmission rate values for each Brazilian state and also took the time-varying reproduction number into consideration.^16^ We used data from official reports on symptomatic infections.^19^^,^^20^ The asymptomatic fraction has been estimated variously in different reports,^25^ as follows: 18% on the Diamond Princess ship;^26^ 31% in repatriation flight screening;^27^ and 50%-75% in the Italian village of Vo’Euganeo.^28^ The average infection lethality ratio and the percentage of symptomatic cases requiring hospitalization were obtained from recent studies^25^^,^^29^ and were adjusted for each Brazilian state according to its demographic pyramid.^30^

The percentage of symptomatic cases requiring hospitalization was estimated from the confirmed cases of SARS-CoV-2 infection in each state,^31^^–^^56^ taking its demographic pyramid and the estimated incidence for each age group into account.^25^ Thus, we estimated that 6.6% to 7.7% (95% confidence interval, CI) of the cases of symptomatic infection in Brazil would require hospitalization.

The average proportion of the patients requiring critical care in an ICU was estimated to be 26.11% of the hospitalized cases, considering recent experience.^25^ We used the numbers of ICU and clinical beds for the month of April 2020 obtained from official Brazilian data sources.^57^ According to general bed utilization reports from before the pandemic,^58^ 34% of clinical beds and 21% of ICU beds were available for patients infected with COVID-19. The input data is presented in **Table 1**.

**Table 1:** Model parameters for each Brazilian state

States	Population	Social distancingindex (%)	Basic reproductionnumber	Hospital ratio ofsymptomatic cases	ICU beds	Clinical beds
Acre (AC)	894,47	47.36	2.20	0.0559	48	765
Alagoas (AL)	3,351,092	43.29	2.06	0.0802	299	3,382
Amazonas (AM)	4,207,714	43.94	2.81	0.0729	271	3,299
Amapá (AP)	861,773	50.78	3.27	0.0606	46	609
Bahia (BA)	14,930,424	41.80	2.20	0.0681	1,478	17,737
Ceará (CE)	9,187,886	47.83	2.85	0.0920	802	11,144
Distrito Federal (DF)	3,052,546	36.40	2.30	0.0633	917	4,388
Espírito Santo (ES)	4,064,052	39.20	2.44	0.0705	716	5,338
Goiás (GO)	7,116,143	36.40	1.96	0.0695	1,053	10,497
Maranhão (MA)	7,114,598	41.60	2.75	0.0804	572	8,23
Minas Gerais (MG)	21,292,666	38.00	1.82	0.0774	3,096	27,87
Mato Grosso do Sul (MS)	2,809,394	37.20	1.88	0.0556	352	3,499
Mato Grosso (MT)	3,526,220	37.50	2.01	0.0581	592	4,773
Pará (PA)	8,690,745	43.10	2.99	0.0842	609	8,448
Paraíba (PB)	4,039,277	48.68	2.61	0.0715	2,006	17,163
Pernambuco (PE)	9,617,072	52.62	2.43	0.1117	454	4,937
Piauí (PI)	3,280,697	44.59	2.02	0.0704	1,408	13,191
Paraná (PR)	11,516,840	43.07	1.83	0.0761	227	4,432
Rio de Janeiro (RJ)	17,366,189	46.56	1.99	0.0970	3,978	20,594
Rio Grande do Norte (RN)	3,534,165	43.95	1.92	0.0715	431	4,497
Rondônia (RO)	1,796,460	45.97	2.35	0.0604	231	2,869
Roraima (RR)	631,181	44.28	2.80	0.0503	25	854
Rio Grande do Sul (RS)	11,422,973	46.37	1.83	0.0740	1,63	19,971
Santa Catarina (SC)	7,252,502	42.74	1.95	0.0653	843	10,541
Sergipe (SE)	2,319,032	40.61	2.31	0.0554	241	2,149
São Paulo (SP)	46,289,333	45.86	1.94	0.0882	8,324	54,698
Tocantins (TO)	1,590,248	41.04	2.05	0.0545	125	2,123

ICU = intensive care unit.

## RESULTS

**Figure 2:**
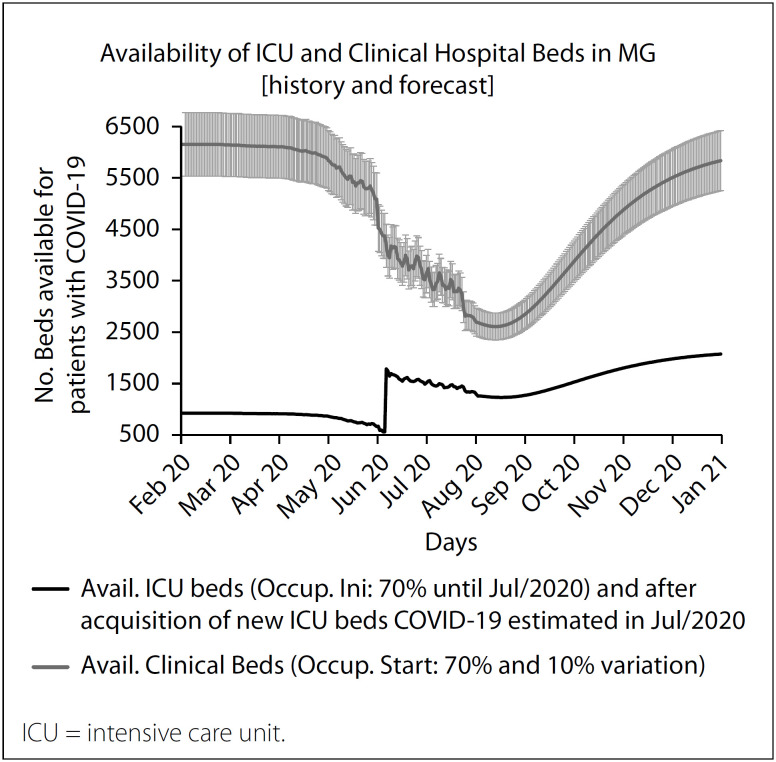
Intensive care unit and clinical bed model for the state of Minas Gerais (MG).

The model considers two different periods of the pandemic: May and August 2020. It uses the historical records of COVID-19 cases^59^ to estimate future infections for each state and project the ICU and clinical bed use for a period of 365 days. **Figure 2** shows an illustrative example of a forecast for August 2020, for the state of Minas Gerais. The model periodically adjusts to the real number of cases, thus providing a fair estimate of future cases of infection. Historical records of infection and future estimates feed the dynamic bed model, which forecasts a reduction in ICU and clinical bed capacity of the state healthcare system. In May 2020, the ICU bed capacity for Minas Gerais was different from the capacity in August 2020. Thus, we adopted a simplified assumption considering a single capacity increase in July 2020. The monitoring of bed procurement did not form part of the scope of the present study.

Accordingly, the model forecasts the possibility of healthcare collapse, i.e. 100% utilization of ICU and clinical beds. Thus, the demand for ICU and clinical beds is established at the peak of infections, since the proportion of symptomatic cases requiring hospitalization produces the peak of capacity utilization of ICU and clinical beds. Although the maximum utilization of bed capacity occurred in May, June and July 2020, the estimate of SARS-CoV-2 infection levels continues to grow in the projection, which produces shortages of ICU and clinical beds for the states. Overall, the results suggest that 0.81% of the population had become infected by May 2020 and that this percentage was 2.87% by August 2020, excluding deaths and individuals who had recovered. Therefore, the majority of the population remains potentially vulnerable. **Table 2** presents the results from the model projection of peak bed utilization and the demand for new hospital beds.

**Table 2:** Model projection of peak bed utilization and demand for new hospital beds

States	Peak ICU bedutilization	Peak clinical bedutilization	ICU bed demand	Clinical beddemand	SUS provision
Acre (AC)	100% (June)	85% (June)	63	142	50 (24%)
Alagoas (AL)	100% (June)	100% (July)	162	286	232 (52%)
Amazonas (AM)	100% (May)	100% (June)	166	293	199 (43%)
Amapá (AP)	100% (May)	100% (June)	38	91	32 (25%)
Bahia (BA)	100% (July)	100% (July)	715	463	1000 (85%)
Ceará (CE)	100% (May)	81%	767	1053	749 (41%)
Distrito Federal (DF)	74%	92%	0	0	337
Espírito Santo (ES)	100% (June)	100% (June)	137	156	636 (217%)
Goiás (GO)	71%	93%	0	0	546
Maranhão (MA)	100% (May)	100% (June)	337	24	390 (108%)
Minas Gerais (MG)	100% (August)	100% (August)	954	1133	1461 (70%)
Mato Grosso do Sul (MS)	71%	85%	0	0	306
Mato Grosso (MT)	72%	88%	0	0	426
Pará (PA)	100% (May)	100% (June)	467	852	376 (29%)
Paraíba (PB)	100% (June)	100% (June)	452	1851	277 (12%)
Pernambuco (PE)	100% (May)	85%	329	0	925 (281%)
Piauí (PI)	100% (July)	100% (July)	282	506	356 (45%)
Paraná (PR)	71%	84%	0	0	818
Rio de Janeiro (RJ)	100% (June)	100% (July)	516	2914	941 (27%)
Rio Grande do Norte (RN)	100% (August)	84%	78	0	272 (349%)
Rondônia (RO)	100% (June)	81%	85	0	152 (179%)
Roraima (RR)	100% (May)	78%	24	0	35 (146%)
Rio Grande do Sul (RS)	100% (July)	100% (July)	841	686	1006 (66%)
Santa Catarina (SC)	100% (July)	91%	151	0	988 (654%)
Sergipe (SE)	100% (May)	100% (June)	95	221	166 (53%)
São Paulo (SP)	100% (June)	100% (July)	2014	2572	3807 (83%)
Tocantins (TO)	100% (May)	100% (June)	196	659	99 (12%)

ICU = intensive care unit; SUS = Sistema Único de Saúde (Brazilian National Health System).

The outcomes show that the Brazilian states require 22,771 additional beds, of which 8,869 (38.95%) are ICU beds, and 13,902 (61.05%) are clinical beds. Populous states, like São Paulo, Rio de Janeiro and Minas Gerais account for nearly 40% of the projected demand.

## DISCUSSION

Our method projected in advance the lack of ICU and clinical beds from May to July 2020. From March to April 2020, technical notes and print and digital media^60^ warned about the estimated maximum utilization rate of public and private hospitals’ bed capacity. These warnings were borne out by reality in most states, as also shown through the model's projections.

These findings provide evidence that SARS-CoV-2 transmission in Brazil is not under control, and the number of active cases remains stable or is even growing in some states, despite the physical distancing policies adopted so far. This suggests that further action is required to prevent higher rates of mortality. Overall, these findings have filled an important gap in estimating the deficit of ICU and clinical beds within the Brazilian healthcare system, across the country. Furthermore, our study also provides a flexible tool that allows healthcare decision-makers to forecast the impact of the pandemic impact and to implement policies for reducing COVID-19 mortality.

The model was implemented in the Python software (Python Language Reference, version 3.8.2, available from http://www.python.org; Python Software Foundation, Amsterdam, 1995). The programming routine automatically captures historical and updated data on COVID-19 cases from a web-based repository that aggregates official data from all states and municipalities that present confirmed cases. The initial model^61^ evolved from a simplified estimate on a spreadsheet to a sophisticated approach embedded in a web-based system that captures data, runs models and displays the results.^59^

The web-based system can be accessed online at https://labdec.nescon.medicina.ufmg.br/webcovid19/. It provides valuable up-to-date information for healthcare managers in advance, which is very useful because acquiring a large number of ICU and clinical beds in a short period is difficult.

The government has implemented a hospital bed census and has obtained additional ICU and clinical beds. Up to November 2020, the Brazilian Ministry of Health had ordered 16,582 beds from suppliers. The cost of these additional beds will be US$ 437 million or R$ 2.34 billion.^62^ Although the Ministry of Health has provided 73% of the overall projected demand, this provision of 16,582 hospital beds differs from (and is lower than) the projected demand for beds at the state level, as presented in **Table 2**. Meanwhile, during pandemics, we suggest that temporary hospitals should be provided, with the supplementary numbers of ICU and clinical beds for each state. This should incorporate use of NPIs, including physical distancing and personal protection policies^63^ such as use of masks, in order to avoid overloading the healthcare system.

In Brazil, physical distancing policies have reduced the intensity of transmission, thereby contributing towards saving many lives. However, in many states, the number of COVID-19 cases remains stable or is even rising, which indicates that distancing control policies should be intensified rather than loosened. Experiences have provided evidence that physical distancing policies have reduced SARS-CoV-2 infection rates,^24^ and have also provided evidence that person-to-person activities have increased the SARS-CoV-2 infection rate.^64^ These findings have demonstrated that economic, cultural, educational or social events involving proximity are dangerous during the COVID-19 pandemic. We identified certain limitations in our study that provide opportunities for future research. Our model is deterministic and assumes discrete values and a uniformly spread population; therefore, a geographical analysis on the spread of the virus is urgently needed in order to determine location-based transmission rates. Additionally, we used an average physical distancing index because physical distancing policies in several states were activated and deactivated at various times. One promising approach for future investigations may comprise formulation of a stochastic model with a multiperiod physical distancing index.

## CONCLUSION

We proposed an integrated approach towards evaluation of the impact of the COVID-19 pandemic on the healthcare system capacity of Brazilian states, through estimating the demand for ICU and clinical beds for each state. The integrated model was applied to two periods of the pandemic for each state and it showed that healthcare would collapse at different times if bed demand, estimated as 22,771 beds, were not satisfied. The government has provided 16,582 hospital beds for the Brazilian states; however, the numbers of beds diverges from the estimated demand projections at the state level.
